# Recent Advances in Experimental Dendritic Cell Vaccines for Cancer

**DOI:** 10.3389/fonc.2021.730824

**Published:** 2021-09-23

**Authors:** Ivan Y. Filin, Kristina V. Kitaeva, Catrin S. Rutland, Albert A. Rizvanov, Valeriya V. Solovyeva

**Affiliations:** ^1^ Institute of Fundamental Medicine and Biology, Kazan Federal University, Kazan, Russia; ^2^ Faculty of Medicine and Health Science, University of Nottingham, Nottingham, United Kingdom

**Keywords:** immunotherapy, cancer, antitumor vaccines, dendritic cells, antigen-presenting cells

## Abstract

The development of immunotherapeutic methods for the treatment of oncological diseases have made it possible to improve the effectiveness of standard therapies. There was no breakthrough after first using of personalized therapeutic vaccines based on dendritic cells in clinical practice. A deeper study of the biology of dendritic cells, as well as the use of new approaches and agents for antigenic work, have made it possible to expand the field of application of dendritic cell (DC) vaccines and improve the indicators of cancer patients. In addition, the low toxicity of DC vaccines in clinical trials makes it possible to use promising predictions of their applicability in wider clinical practice. This review examines new approaches and recent advances of the DC vaccine in clinical trials.

## Introduction

**Figure 1:**
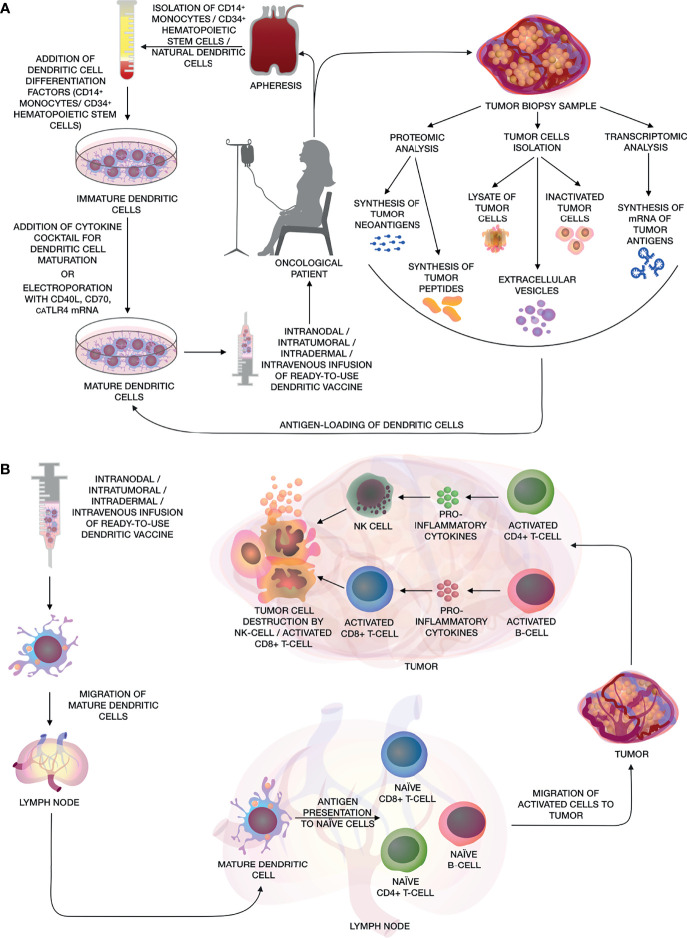
**(A)** The main stages of creating a personalized antitumor vaccine based on autologous DCs. The figure shows various methods for obtaining DCs from CD14^+^ monocytes, CD34^+^ HSCs and natural DCs from patient’s whole blood by apheresis, further maturation using a cocktail of cytokines or electroporation of mRNA of CD40L, CD70 and caTLR4. Methods for obtaining various types of tumor antigens are also shown, such as: artificial neoantigens/peptides/mRNA of tumor antigens, lysate/inactivated tumor cells, as well as extracellular vesicles for activating DCs and their subsequent use as a DC vaccine. **(B)** After the administration of the dendritic vaccine, activated mature DCs migrate to the lymph nodes, where they present tumor antigens to naïve CD8^+^ and CD4^+^ T-cells and B-cells. Activated CD8^+^ and CD4^+^ T-cells and B-cells migrate to the adjacent tumor tissue, where tumor cells are eliminated by activated cytotoxic CD8^+^ T-cells and NK cells, while CD4^+^ T-helpers and B-cells releasing pro-inflammatory cytokines, that enhance cytotoxic effects.

Immunotherapeutic methods have opened up new possibilities for the treatment of cancer. Therapeutic anticancer vaccines, in particular DC vaccines, have played a significant role in the development of these methods (1). The mechanism of action of DC vaccines is to stimulate and support an immune response aimed exclusively at the elimination of tumor cells in the body. Dendritic cells (DCs), along with macrophages and B-cells, have major histocompatibility complex (MHC) -II molecules on their membrane surface which make them professional antigen-presenting cells (APCs). But only DCs, due to their ability to migrate to the lymph nodes, have the ability to activate T-killers – the main cells in the antitumor immune response (2, 3). The general approach for creating DC vaccines is to isolate dendritic cells or their precursors from human blood, further maturation and activation of cells using a cytokine cocktail and autologous tumor antigens *ex vivo*, followed by administrating of autologous mature activated DCs back into the body (**Figure 1**). Once in the lymph nodes, DCs carry out antigen presentation for CD4^+^ and CD8^+^ T-cells, activating the cell-mediated and humoral adaptive immune system. Moreover, these vaccines can act as a safe adjunct to complex anticancer therapy, thus enhancing the therapeutic effects of chemotherapy (4) or other immunotherapeutic methods such as immune checkpoint inhibitors (ICIs) (5). Sipuleucel-T – the first FDA-approved immunotherapeutic drug based on autologous DCs treated with the recombinant prostatic acid phosphatase (PAP) and granulocyte-macrophage colony-stimulating factor (GM-CSF) fusion, has shown not only no toxic effects, but also positive prospects in therapeutic approaches for the treatment of prostate cancer (6). Sipuleucel-T is currently being tested in clinical trials in combination with other anticancer therapies (NCT01804465, NCT02463799, NCT01881867). Nowadays, after numerous clinical trials, DC vaccines have shown low efficacy in the treatment of solid and hematological tumors. However, DC vaccines in combination with other anticancer therapy can be hopefully become a tool, aimed at organizing a targeted antitumor T-cell response.

## Methods of Obtaining Dendritic Cells

Nowadays, there is no single optimal method to generate DCs. Under culture (*ex vivo*) DCs can be obtained in two ways: differentiation of DCs from peripheral blood monocytes or from CD34^+^ hematopoietic stem cells (HSC). Cytokines such as GM-CSF and IL-4 are required for differentiation of immature DCs from monocytes derived from human peripheral blood. Using this method, the process of obtaining immature moDCs takes 5-7 days. After that, immature DCs are stimulated with a cytokine cocktail (IL-1β, IL-6, TNF-α or CD40L and prostaglandin (PGE)-2 or TLR agonists) for 48 hours to produce a mature moDC (7).

In addition, mRNA can be used for DC maturation. One study used mRNAs encoding proteins CD40L, CD70, and constitutively active TLR4 (caTLR4) (TriMix-DC), which were electroporated into DCs. TriMix-DCs that were given to all patients were characterized by a mature phenotype and secretion of IL-12p70 (8). There is also a method for culturing polarized α-type 1 DCs (αDC1), which are capable of producing high levels of IL-12 and inducing long-lived type 1 T-cell responses against tumor associated antigens more efficiently than standard mature DCs. The following cytokine cocktail for DCs maturation was used: IL-1β, TNF-α, IFN-α, IFN-γ and polyinosinic:polycytidylic acid (poly-I:C) (9).

Another method of obtaining DCs from CD34^+^ HSCs, derived from bone marrow by administering GM-CSF to patients prior to leukapharesis which involves culturing the harvested cells for 1 week in the presence of GM-CSF, TNF-α, and recombinant fms-like tyrosine kinase 3 ligand (Flt3L). As a result, DCs are obtained, which are phenotypically similar to Langerhans and myeloid cells at different stages of differentiation. Such CD34-HSCs-derived DCs also have an important influence on the T-cell response as they can induce a cellular or humoral immune responses depending on their differentiation into either LCs or dermal CD14^+^ DCs (10, 11).

There is also a method for generating circulating DCs *in vivo* by administering growth factors for hematopoietic cells like Flt3L or G-CSF (12). Some studies use natural myeloid DCs to create a DC vaccine. The main advantage of using natural DCs instead of moDCs is that the short-term *ex vivo* exposure needed to activate cells and load them with tumor antigens, usually it takes less than 24 hours. This advantage can preserve their functionality better and prevent depletion. However, their numbers can be significantly lower than moDCs or CD34-HSCs-derived DCs (13).

## Methods for Activating Dendritic Cells

Various methods are currently being used to activate DCs *in vivo* in order to overcome suppression of the immune system by tumors. TLRs are the main class of cellular receptors responsible for recognizing foreign microorganisms or viruses. Synthetic agonists of the TLR receptors can influence the depth of antitumor T-cell responses. TLR7 agonists such as imiquimod, for example, stimulated immune-mediated rejection of primary skin metastases when it was applied topically in breast cancer patients (14). Poly-I:C is another antitumor therapy of interest as it is an immunostimulating agent that interacts with TLR3 and thereby induces the maturation of DCs, as well as the production of IL-12, type I IFNs and chemokines. A less toxic variant, poly-ICLC, is a poly-I:C mixed with the stabilizers carboxymethylcellulose and polylysine. It is used either alone or as an adjuvant to the main anticancer therapy (15). In one recent multicenter randomized phase II trial, patients were divided into two groups and vaccinated with CDX-1401 (human anti-DEC-205 mAb) + poly-ICLC, with or without CDX-301 (Flt3L) pretreatment (NCT02129075). Patients in the group receiving Flt3L showed increased numbers of activated DCs, natural killers (NK) and T-cells, but also exhibited an increase in T-cell responses, which increased the effectiveness of the vaccine. Furthermore, poly-ICLC enhanced the antigen-presenting activity of DCs (16).

There have also been attempts to enhance antitumor effects by combining DC vaccines with a lentiviral vector encoding anti-TGF-β1 shRNA (shTGF-β1) (17). Lentivirus (LV) was injected into mouse tumors the day before DC vaccine administration. However, the combination of LV shTGF-β1 with the DC vaccine was insufficient for long-term tumor suppression of TGF-β1 (18).

In order to obtain a complete DC vaccine, autologous mature DCs obtained from mononuclear cells or CD34^+^ HSC are activated *ex vivo*. The process of antigenic loading of DCs *ex vivo* is performed in various ways including *via*: inactivated tumor cells (19), tumor lysate (20), tumor vesicles (21, 22), synthesized tumor peptides (13) or synthesized mRNA of tumor antigens (8).

## Clinical Trials

### Carcinoma

The search for approaches towards developing and testing DC-based vaccines continues, despite the difficulties associated with low efficacy (**Table 1**). It was shown that a vaccine composed of monocyte-derived autologous Th17-inducing DCs pulsed with folate receptor alpha (FRα) epitopes was highly immunogenic and promoted the prolongation of the remission period in patients with ovarian cancer. At the same time, no adverse effects (AEs) at grade 3 or higher were found (28). In a study by Nagai et al., an adherent population of peripheral blood mononuclear cells was used to create a vaccine based on DCs. These cells were stimulated by a number of factors to produce mature DCs, and then pulsed with Wilms tumor gene 1 (WT1) peptide and Mucin 1 (MUC1), this vaccine was shown to be effective in a Phase I/IIa Clinical Trial in patients with adenocarcinoma without AEs of grade 2 or higher (29). The safety of DC-based vaccines has been demonstrated in patients with hepatocellular carcinoma using HSP70 mRNA-transfected DCs. 75% of the patients had AEs of grade 1 or 2, however, the incidence of all AEs between the control and DC groups were comparable, since most AEs were associated with liver disease. In the DC group, 10% of patients had AEs of grade 3, which included anemia and thrombocytopenia (30).

**Table 1:** Effectiveness of personalized therapeutic DC vaccine in combination with other antitumor therapy methods.

Combination therapy	Cancer type	Efficiency, (%)	Ref.
PD-1 inhibition monotherapy after DC vaccination	Metastatic melanoma	CR=24, PR=28, ST=21, PD=28, mPFS=13.1 (months), mOS=32.5 (months)	(5)
Ipilimumab monotherapy after DC vaccination		CR=20, PR=15, ST=15, PD=50, mPFS=3.9 (months), mOS=30.0 (months)	
Ipilimumab- nivolumab after DC vaccination		CR=25, PR=50, PD=25, mPFS=5.6 (months), mOS= NR	
TriMixDC-MEL with ipilimumab	Melanoma	CR=20, PR=18, mPFS=27 (weeks), mOS=59 (weeks)	(23)
DC vaccination with cisplatin	IV stage melanoma	mPFS=4.7 (months), mOS=12.2 (months)	(24)
DCVax-L with radiotherapy and temozolomide	Glioblastoma	mOS=23.1 (months)	(25)
DC vaccination in combination with low-dose cyclophosphamide, poly I:C, imiquimod and anti-PD-1 antibody.	Glioblastoma multiforme	mOS=19 (months)	(26)
	NSCLC	mOS=17 (months)	
IFN-DC with low-dose intra-tumoral rituximab	Relapsed/Refractory Follicular Lymphoma	CR=37, PR=12.5, mPFS=13.5 (months)	(27)

*CR, complete response; PR, partial response; ST, stable disease; PD, progressive disease; mPFS, median progression-free survival; mOS, median overall survival; NR, no results; NSCLC, non-small cell lung cancer.

### Melanoma

It has been shown that a vaccine based on moDCs loaded with tumor lysate affects the tumor microenvironment (TME) and promotes the transformation of a “cold” tumor into a “hot” one through activation and infiltration of CD8^+^T-lymphocytes, but it also affects up-regulation of PD-L1 expression in patients with metastatic melanoma (31). A total of 21 patients participated in the trial and the median overall survival (OS) was 22 months. One patient achieved a complete response (CR), two had partial responses (PR), and nine patients were classified as having a stable disease (SD). The aspect of the change in the expression of PD-L1 of tumor cells after the application of the DC vaccine, on the one hand, indicates the activation of the tumor “escape” processes, but, on the other hand, makes it possible to include an effective additional immunotherapy using ICIs in the treatment process (32). The combination of a DC vaccine with ICIs has been shown to be effective. 51 melanoma patients treated with ICIs following recurrence had an adjuvant DC vaccination. The response rate with pembrolizumab or nivolumab alone was 52%, and 35% responded to ipilimumab monotherapy. The effectiveness of ipilimumab in combination with nivolumab was 75% (5).

A vaccine based on moDCs electroporated with mRNA encoding CD40 ligand, CD70 and a caTLR4 (TriMixDC) has also been tested in melanoma patients. Effective antigenic loading was achieved by coelectroporation of TriMixDC with full-length mRNA encoding HLA-II proteins and melanoma-associated genes (MAA): MAGE-A3, MAGE-C2, tyrosinase or gp100, (TriMixDC-MEL), which allows presentation of the full spectrum of antigenic peptides and leads to a broader MAA-specific T-cell response. CR was observed in two out of 15 patients, and PR was also observed in two patients. Median progression-free survival (PFS) and OS were 5 months (95% confidence interval (CI), 0 to 10) and 14 months (95% CI, 5 to 23), respectively. No serious grade 3 or 4 toxicity was observed, however all patients had grade 2 local skin reactions (irritation, erythema and edema) at the injection sites. In addition, post-infusion grade 2 chills were observed in 3 of 15 patients (8).

In phase II clinical trials, in contrast to the previous study, in addition to TriMixDC-MEL, therapy with checkpoint inhibitors, in particular, ipilimumab, was used. Among 39 patients treated, 20% were reported with CR and 18% with PR. The estimated median PFS was 27 weeks (95% CI, 9 to 44) and the estimated median OS was 59 weeks (95% CI, 40 to 79). 64% of patients died as a result of a progressive disease. All patients had skin reactions at the injection site of TriMixDC-MEL grade 2, and some patients had chills (grade 1-2) and flu-like symptoms. 36% of patients developed treatment-related grade 3 or 4 AEs. In 64% of patients, grade 1-2 dermatitis was registered. All treatment-related AEs were reversible with the exception of hypopituitarism (23).

TriMixDC-MEL was used also used as an adjuvant in phase II randomized controlled clinical trials for patients with stage III/IV melanoma who did not have the disease after removal of macrometastases. This vaccine has been shown to be well tolerated by patients and to improve patient survival over the course of a year. However, despite the promising results, patients in the TriMixDC-MEL group had a higher rate of early local-regional relapse compared to the control group. No AEs of grade 3 or higher were identified, but 80% of patients reported swelling and erythema at the site of intradermal DC-injection (33). What is more, in a recent prospective, randomized, phase II clinical trial, a DC vaccine in combination with cisplatin in stage III and IV melanoma patients was feasible and safe, but this combination did not improve clinical outcomes when compared to DC vaccination monotherapy (24).

Autologous natural myeloid DCs have also been used to treat melanoma patients. One such study included 15 patients with stage IV or inoperable stage IIIc metastatic melanoma. DCs were loaded directly with HLA-A*0201-restricted melanoma-associated peptides (gp100 and tyrosinase). The mean PFS was 17.6 months for patients with functional T-cells in their blood and skin-infiltrating T-lymphocytes (SKIL) producing IFN-γ. The mean OS of patients with functional T-lymphocytes was 29.0 months. There were no vaccine-related side effects, with the exception of one patient with grade 1 pain at the injection site and 4 patients with grade 1 fatigue (13).

A DC vaccine with natural myeloid DCs loaded with peptides is currently in a clinical trial for the treatment of stage 3 melanoma patients (NCT02993315). In addition, patients are still being recruited for phase I clinical trials of a personalized vaccine created by autologous DCs treated with autologous whole tumor cell lysate in combination with the chemotherapy drug cyclophosphamide to treat advanced solid tumor patients with high tumor mutation burden (NCT03671720).

### Glioma

Glioblastoma multiforme (GBM) is one of the most common primary malignant brain tumors and is characterized by low patient life expectancy, “inaccessibility” for activated T-cells, and high genetic variability (34). It was shown that after the use of a DCVax^®^-L vaccine (DCs loaded with autologous tumor lysate), 33% of patients reached or exceeded the median life expectancy (48 months), and 17% of patients reached or exceeded the median life expectancy of 72 months in phase I/II clinical research. No AEs of grade 3 or higher were identified. AEs of grade 1 and 2 included headache, nausea, loss of appetite, diarrhea, fatigue and low-grade fever (35). DCVax^®^-L is currently being tested in a phase III clinical trial in patients after glioblastoma resection in combination with radiation and chemotherapy. Median OS was 23.1 months from surgery, and vaccination-related grade 3 or 4 AEs were observed in only 2.1% of patients (25).

A study conducted on patients with high-grade glioma showed encouraging results in a phase II clinical trial using α-type-1 DCs pulsed with a cocktail of 5 synthetic peptides derived from enriched monocytes in the presence of a cytokine cocktail (36).

In a study by Wang et al. DCs loaded with tumor-associated antigens (TAA) and/or mRNA of neoantigens have been used in a small group of glioma patients in combination with low-dose cyclophosphamide to regulate T-cell depletion, poly-I:C, and imiquimod. As a result of this study, it was shown that loading DCs with TAA and the mRNA of neoantigens increased the life expectancy of patients. The median OS was 19 months and no grade 3 or higher AEs were observed (26).

### Sarcoma

A therapeutic vaccine based on moDCs loaded with antigens from a patient’s tumor has been shown to be safe in phase I/II academic clinical trials in pediatric patients with high-risk metastatic tumors, mainly sarcomas and neuroblastomas. Besides these outcomes, the study showed that certain combinations of anticancer drugs (temozolomide + irinotecan), as well as a combination (pazopanib + topotecan + cyclophosphamide), prior to cell isolation in patients, negatively affected the maturation of DCs and reduced their immunostimulating properties (37). Сombinations of DCs loaded with tumor lysate with imiquimod (Aldara^®^) and gemcitabine (Gemzar^®^) in sarcoma patients are now in active phase (NCT01803152). Favorable results of DC testing were obtained in young patients and children with soft tissue sarcoma. Intratumoral administration of DCs, in combination with radiation doses and subsequent tumor resection, resulted in the absence of systemic relapses in 11 out of 18 patients within 2-8 years. There were no grade 4 or 5 toxicities and no treatment-related deaths were observed. The median OS was 57 months (38). Patients with recurrent or refractory Ewing’s sarcoma who received intradermal vaccine administration based on moDC loaded with tumor lysate did not have a pronounced clinical response, however, some of them showed activation of the T-cell immune response. Probably disappointing clinical response data are associated with prior treatment, which had a serious immunosuppressive effect, as well as advanced disease and extensive lesions (39).

### Lymphoma

Hematologic malignancies are of particular interest for DC-based therapies, as they usually present with multiple areas of immune dysregulation. In a study by Cox et al. moDCs generated in the presence of IFN-α and GM-CSF (IFN-DC), in combination with low doses of rituximab, were administered intranodally in a phase I clinical study in patients with follicular lymphoma. Interestingly, even in the absence of “loading” these cells with TAA, impressive immunological responses were recorded in some patients and regression of some areas not undergoing therapy. No grade 3 or higher AEs were observed (27). In another study immunological responses to anti-PD-1 antibodies (pembrolizumab) were evaluated in combination with the intranodal administration of the Lymvac-1 vaccine in patients with follicular lymphoma (NCT02677155). A clinical trial in non-Hodgkin’s lymphoma patients with DCs in combination with pembrolizumab and cryosurgery is presently in the active phase (NCT03035331). An earlier similar clinical trial, but using just intratumoral DCs and cryoablation, has already been shown to be safe for patients (NCT01239875) (40).

## Conclusion

The first attempts at DC-based vaccines were made over 20 years ago, but the results have not been encouraging. Researchers faced particular difficulties while DC vaccine was being developed. Differentiation antigens or overexpressed tumor antigens were used as the target antigen. However negative selection in the thymus limits the formation of cytotoxic T-lymphocytes with high avidity directed against these antigens. In order to avoid this problem, scientists identified neoantigens recognized by tumor infiltrating lymphocytes (TILs), and certain adjuvants increased the immunostimulatory effect and activation of DCs. Though, tolerogenicity and dysfunction of DCs in the TME became the main problems with DC vaccines. It is important to improve our knowledge regarding regulating these processes. The accumulation of knowledge about the biology of DCs, as well as the development of new approaches towards treating malignant neoplasms, have allowed scientists to improve existing technologies and increase the effectiveness of developments based on DCs. Thus, despite their limited effectiveness, DC vaccine technology is promising in relation to aspects linked to personalized medicine, clinical safety and is most effective in combination with other therapeutic approaches. In addition, it should be borne in mind that the majority of patients receiving experimental DC-based drugs were in the terminal stages of the disease. In summary, DC vaccines in combination with another immunotherapeutic or traditional anticancer methods inspire hope for the treatment of patients at earlier disease development stages or to delay or prevent relapse and metastasis, as DC vaccines have exhibited an increase in the survival rate of responded patients with advanced stages of oncological disease, and also had a significant effect on the destruction of distant metastatic foci.

## Author Contributions 

Conceptualization, AR. and VS. Writing—original draft preparation, IF, KK and VS. Writing—review and editing, AR and CR. Visualization, KK. Supervision, AR. All authors contributed to the article and approved the submitted version.

## Funding

The work was performed according to the Russian Government Program of Competitive Growth of Kazan Federal University.

## Conflict of Interest

The authors declare that the research was conducted in the absence of any commercial or financial relationships that could be construed as a potential conflict of interest.

## Publisher’s Note

All claims expressed in this article are solely those of the authors and do not necessarily represent those of their affiliated organizations, or those of the publisher, the editors and the reviewers. Any product that may be evaluated in this article, or claim that may be made by its manufacturer, is not guaranteed or endorsed by the publisher.
